# Sleep Disturbance and Related Factors in Patients with Nasopharyngeal Carcinoma and Their Family Caregivers Prior to the Initiation of Treatment

**DOI:** 10.1038/s41598-018-32587-9

**Published:** 2018-09-24

**Authors:** Xiao-Ying Lai, Zhong-Min Tang, Xiao-Dong Zhu, Ling Li, Xue-Yan Qin, Jiang-Ling Lan, Chun-Ping Lu, Zhi-Chan Lyu, Li-Qiao Liang, Li-Jun Chen

**Affiliations:** 1 Department of Radiation Oncology, Cancer Hospital of Guangxi Medical University, Cancer Institute of Guangxi Zhuang Autonomous Region, Nanning, China; 2 Department of Quality Control, Cancer Hospital of Guangxi Medical University, Cancer Institute of Guangxi Zhuang Autonomous Region, Nanning, China

## Abstract

Sleep disturbance is a common complaint in cancer patients. However, less is known about the parameters of sleep in patients with nasopharyngeal cancer (NPC) and their family caregivers (FCs) when they are about to begin treatment. We investigated the sleep quality in patients with NPC and their FCs before treatment and determined the related factors that predict sleep disturbance in these patients before therapy. A total of 101 patient-FC dyads were recruited. They completed the Pittsburgh Sleep Quality Index (PSQI) prior to treatment. No differences were found in sleep disturbance between patients (38.6%) and their FCs (31.7%). Patients reported significantly higher rates of short sleep duration than their FCs (P = 0.011). Logistic regression analyses showed that older patients were more prone to suffer from poor sleep quality before treatment (OR = 1.06, 95% CI = 1.01–1.10, P = 0.008), while patients with a higher BMI were less likely to experience sleep disturbance (OR = 0.83, 95% CI = 0.71–0.96, P = 0.012). Sleep disturbance is a significant problem in patients with NPC and their FCs before therapy. Older patients and those with a lower BMI appear to be more inclined to suffer from poor sleep before treatment.

## Introduction

Nasopharyngeal carcinoma (NPC) is highly prevalent in Southeast Asia and southern China^1^. With the widespread use of intensity-modulated radiotherapy (IMRT) combined with platinum-based chemotherapy (induction chemotherapy, concurrent chemotherapy, or adjuvant chemotherapy), patients with NPC have presented excellent long-term results. The 5-year survival rate has come close to 85%^2^. Along with the favourable outcomes of NPC, the side effects of radio-chemotherapy and quality of life after treatment have received considerable attention.

Sleep disturbance is a common complaint in cancer patients. A previous report showed that the occurrence of clinically significant levels of sleep disturbance in oncology patients at the initiation of treatment ranged between 26% and 57% based on the Pittsburgh Sleep Quality Index (PSQI) measures^3,4^. This rate is higher than that in the general population^5^. These sleep problems can present before and after diagnosis of cancer and may persist during and/or after the completion of treatment^6^. Poor sleep quality is correlated with negative health outcomes, including decreased psychological and physical functioning, and a worse quality of life^4,7,8^. Moreover, sleep disturbances may result in unplanned treatment interruptions, a lower tolerance for treatment, or a change in the treatment regimen and may even be linked to adverse events and a worse prognosis^9^.

Family caregivers (FCs) of patients with cancer are also at risk of developing sleep disturbance. FCs perform an indispensable role in providing health care to cancer patients, such as managing their symptoms, providing them with emotional care, and coordinating various care-related tasks. Several investigations have suggested that between 36.7% and 76% of caregivers report mild to severe sleep disturbance^10,11^, which may negatively influence their emotional well-being^12^, quality of life^13^, and ability to provide care to the patients.

However, the majority of sleep studies have included patients with a variety of cancer diagnoses^3,4,14^. In addition, most of the subjects in studies conducted to examine sleep disturbances have been patients with breast cancer, prostate cancer, lung cancer, or brain tumours^3,4,14^. More research that focuses on a specific cancer diagnosis is needed to discern whether sleep patterns are the same in patients with different forms of cancer and to identify the factors that predict sleep disturbance in these patients. Although there has been some research evaluating sleep disorder in patients with NPC, these studies were nearly all focused on obstructive sleep apnoea syndrome^15,16^. Few studies have examined the sleep parameters of patients with NPC^17,18^. In one of the first studies of sleep disturbances in patients with NPC, Qin *et al*.^17^ noted that 36.5% of patients reported sleep problems before the initiation of cancer treatment. However, the proportion of patients who displayed worse sleep quality significantly increased after radiotherapy (RT) and concurrent chemotherapy. In another study of 51 patients with NPC^18^, patients with poor sleep quality prior to RT comprised 37.3% of the subjects. Nearly 64.7% of patients experienced lower sleep quality after RT. Additional information on sleep parameters is warranted in patients with NPC before treatment because of the high prevalence and negative consequences of sleep disturbances in these patients. Furthermore, poor sleep quality before treatment was likely to correlate with worse sleep in patients with NPC after therapy^18^.

Certain demographic characteristics and other risk factors may correlate with subjective and objective sleep disturbances in patients. While a previous study reported that sleep dysfunction was associated with younger age^19^, in patients with NPC, age was found not to correlate with poor sleep quality^18^. Similarly, while a higher body mass index (BMI) has been correlated more with objective sleep disturbance at the initiation of RT^20^, a study of predictors of sleep disturbance found no association between BMI and sleep disturbance^21^. Along with these inconsistencies in risk factors for sleep disturbance in general, even less is known about the parameters of sleep in patients with NPC and their FCs about to begin treatment. Therefore, the objectives of this study were to describe the prevalence of clinically significant sleep disturbance in patients with NPC and FCs prior to the initiation of treatment and to compare the differences in their sleep parameters. The second aim was to determine which factors (demographic and personal characteristics, and cancer-specific variables) predict poor sleep quality in patients with NPC before therapy. We hypothesised that both patients with NPC and their FCs would report worse sleep quality prior to the initiation of treatment.

## Results

### Patient and FC Characteristics

A total of 120 patents with NPC were approached for enrolment in this study. In total, 19 patients (15.8%) and 3 FCs were excluded. Among the excluded patients, 7 had no FC, 5 refused to participate because they were afraid to take the test, and 7 patients were excluded because their FCs did not meet the eligibility criteria. A total of 101 patients were included in the final analysis. The 101 corresponding caregivers were interviewed. Table 1 lists a summary of the demographic characteristics of the 101 patient-FC dyads. Patients were significantly older (P = 0.010) and more likely to be male (78.2%, P = 0.000) than the FCs. Patients were diagnosed with stage II (13.9%), stage III (25.7%), or stage Iva + b (60.4%). The percentage of patients with Karnofsky performance score (KPS) = 90 was 100%, whereas most FCs had a KPS = 100 (98%, P = 0.000). The majority of the FCs were the patient’s spouse or partner (49.5%) or their parent or child (32.6%). There were no significant differences between patients and FCs in terms of BMI and educational level.

**Table 1:** The demographic and clinical characteristics of patients and their family caregivers (N = 101).

Characteristics	Patients N (%)	Caregivers N (%)	P
Age (Mean ± SD)	42.6 ± 10.2	39.0 ± 9.4	0.010
BMI (Mean ± SD)	21.3 ± 3.0	21.7 ± 3.0	0.882
Gender			0.000
Male	79 (78.2)	45 (44.6)	
Female	22 (21.8)	56 (55.4)	
KPS			0.000
90	101 (100)	2 (2.0)	
100	0 (0.0)	99 (98.0)	
Education level			0.056
Primary school	17 (16.8)	13 (12.9)	
Junior school	55 (54.5)	41 (40.6)	
High school	10 (9.9)	21 (20.8)	
College or higher	19 (18.8)	26 (25.7)	
Marital status			0.001
Married	90 (89.1)	70 (69.3)	
Single/divorced	11 (10.9)	31 (30.7)	
Stage (7th UICC)			
Stage II	14 (13.9)		
Stage III	26 (25.7)		
Stage Iva + b	61 (60.4)		
Relationship to patient			
Spouse/partner		50 (49.5)	
Parent/Child		33 (32.6)	
Brother/sister		14 (13.9)	
Other relatives		4 (4.0)	

Abbreviation: SD, Standard Deviation; BMI, Body Mass Index; KPS, Karnofsky Performance Score; UICC, Union for International Cancer Control.

### Differences Between Patients and FCs in the Prevalence of PSQI Global or Subscale Scores Prior to the Initiation of Treatment

The mean global PSQI scores of patients with NPC and FCs prior to the initiation of treatment were 7.2 (SD, 4.3) and 6.5 (SD, 4.1), respectively. A total of 39 (38.6%) patients had global PSQI scores greater than 7, meaning that they suffered from poor sleep quality (Table 2), while 32 (31.7%) FCs reported poor sleep quality. No differences were found between patients and their FCs in the prevalence of clinically significant levels of sleep disturbance on the basis of the cut-off scores for the PSQI global score (P = 0.302).

**Table 2:** The differences of the PSQI scores between patients and caregivers.

PSQI components	Score	Patients		Caregivers		P
		N	%	N	%	
Subjective sleep quality						0.344
	0	16	15.8	25	24.7	
	1	49	48.5	42	41.6	
	2	23	22.8	25	24.8	
	3	13	12.9	9	8.9	
Sleep latency						0.413
	0	20	19.8	21	20.8	
	1	39	38.6	35	34.6	
	2	24	23.8	33	32.7	
	3	18	17.8	12	11.9	
Sleep duration						0.011
	0	42	41.5	64	63.4	
	1	33	32.7	17	16.8	
	2	15	14.9	9	8.9	
	3	11	10.9	11	10.9	
Habitual sleep efficiency						0.377
	0	53	52.5	55	54.5	
	1	19	18.7	26	25.7	
	2	14	13.9	8	7.9	
	3	15	14.9	12	11.9	
Sleep disturbances						0.474
	0	5	5	9	8.9	
	1	67	66.3	69	68.3	
	2	26	25.7	22	21.8	
	3	3	3	1	1	
Use of sleep medications						0.466
	0	95	94	97	96	
	1	2	2	1	1	
	2	2	2	3	3	
	3	2	2	0	0	
Daytime dysfunction						0.600
	0	27	26.7	20	19.8	
	1	34	33.7	38	37.6	
	2	24	23.8	29	28.7	
	3	16	15.8	14	13.9	
Global PSQI score						0.302
	≤7	62	61.4	69	68.3	
	>7	39	38.6	32	31.7	

Abbreviation: PSQI, Pittsburg Sleep Quality Index.

There was no difference between patients and their FCs in the occurrence rates of any of the PSQI subscale scores except for sleep duration before treatment. Patients reported significantly higher rates of short sleep duration than their FCs (P = 0.011). Of note, there was a low rate of use of sleep medications for both patients and their caregivers (6.0% and 4.0%, respectively). Additionally, no statistically significant difference between the two groups was found (P = 0.466).

### Relationships Among the Demographic and Clinical Characteristics and PSQI Global Scores and Subscale Scores of Patients Before Treatment

As shown in Table 3, the PSQI global score and the use of sleep medication of patients before treatment was significantly positively associated with clinical stage. Sleep duration was correlated with gender. Significant correlations were found between sleep efficiency and age, level of education and disease stage. Sleep disturbance was positively associated with age, but negatively correlated with education level. Daytime dysfunction was positively associated with education.

**Table 3:** Correlation between PSQI scores and patients’ demographic and clinical characteristics.

PSQI components	Age	Gender	Marital status	Education level	BMI	Stage
Sleep quality	−0.054	0.053	0.006	−0.028	−0.170	0.192
Sleep latency	0.061	−0.050	0.058	−0.153	−0.072	0.174
Sleep duration	0.129	−0.209*	0.070	−0.133	0.044	0.140
Sleep efficiency	0.218*	−0.172	−0.004	−0.283**	−0.132	0.256**
Sleep disturbance	0.242*	0.015	−0.066	−0.211*	−0.082	0.153
Use of sleep medication	−0.002	−0.031	0.052	−0.057	−0.051	0.198*
Daytime dysfunction	−0.059	−0.029	0.096	0.219*	−0.179	0.145
PSQI global score	0.121	−0.91	0.048	−0.168	−0.174	0.234*

Abbreviation: PSQI, Pittsburgh Sleep Quality Index; *P < 0.05; **P < 0.01.

### Predictors of Poor Sleep Quality in NPC Patients Before Therapy

Logistic regression analyses showed that age and BMI were predictors of poor sleep quality in patients with NPC prior to the initiation of treatment (Table 4). Older patients were more prone to suffer from poor sleep quality before treatment (OR = 1.06, 95% CI = 1.01–1.10, P = 0.008), while patients with a higher BMI were less likely to experience sleep disturbance (OR = 0.83, 95% CI = 0.71–0.96, P = 0.012). None of the other values correlated with poor sleep quality in patients with NPC before treatment.

**Table 4:** The predictors of sleep disturbance in patients before treatment.

Variable	B	Wals	P	OR	95%CI
Age	0.05	7.01	0.008	1.06	1.01–1.10
BMI	−0.19	6.26	0.012	0.83	0.71–0.96

Abbreviation: BMI, Body Mass Index; OR, Odds Ratio; 95% CI = 95% Confidence Interval.

## Discussion

This study is the first to assess the differences in the prevalence of sleep disturbance in patients with NPC and their FCs using subjective measures and to identify the predictors of sleep disturbance prior to the beginning of treatment in these patients. Consistent with our hypothesis, the results showed that the prevalence of sleep disturbance before treatment was similar for both patients and their FCs. However, the patients reported significantly higher rates of short sleep duration than their FCs. Older patients were more prone to suffer from poor sleep quality prior to the initiation of treatment, while patients with a higher BMI were less likely to experience sleep disturbance.

As the favourable outcomes of patients with NPC after treating with RT and chemotherapy, quality of life in these patients have received more and more attention. Sleep disturbance, which has been proven to affect quality of life, is a common symptom observed in oncology patients^22^, including patients with NPC. The results of this study showed that 38.6% of patients had clinically significant levels of sleep disturbance on the basis of global PSQI scores greater than 7 prior to the initiation of treatment, which is higher than the general population^5,23^. This percentage is similar to that reported in previous investigations of patients with NPC, which found sleep disturbance in approximately 37% of patients before treatment^17,18^. However, the cut-off point of sleep disturbance according to the global PSQI scores in these reports was greater than 5, which is lower than the cut-off point (>7) used in our study. Therefore, the proportion of sleep disturbance in our study is higher than those of previous investigations. The reasons for the difference in results may be that our study enrolled more older patients who were more likely to suffer from poor sleep quality before treatment.

There is an increasing body of literature suggesting that FCs are at high risk for sleep disturbance before, during, and after providing care to a family member^24,25^. Our present study showed that 31.7% of the FCs reported global PSQI scores that were higher than the cut point (>7), a prevalence rate that exceeds that of the general population^5^. To our knowledge, this study is the first to explore the prevalence of sleep disturbance in a sample of FCs of patients with NPC. Very little information is available on the level of sleep problems experienced by FCs. However, a previous study investigating FCs of patients with prostate cancer showed that the prevalence of sleep disturbance in FCs was 36.7% before initiating radiation therapy^10^, which is similar to our result. Conflicting results were reported in a study of FCs of patients with other cancer diagnoses (breast, prostate, lung, brain cancer), in which rates of sleep disturbance were between 40% and 50% at the initiation of RT^26^. Carter *et al*.^27^ even found that 95% of the FCs of patients with advanced cancer reported severe sleep problems. Possible reasons for these inconsistencies may be related to the methods used to categorise the sleep disorder, the cancer stage, and differences in patients’ cancer diagnoses. The prevalence of sleep disturbance in FCs of patients with a variety of cancer diagnoses may be different. Further investigation of specific cancer diagnoses is warranted to determine the sleep characteristics in patients with different forms of cancer. We found no significant differences between patients and their FCs in the occurrence rates of sleep disturbance based on the PSQI global score, which is consistent with most findings from previous studies^26,28^.

In the current study, 58.5% of patients slept less than 7 hours (score >0) before treatment, while this rate was only 36.6% in FCs. Thus, patients reported significantly higher rates of short sleep duration than their FCs. This result is inconsistent with a study by Carney, who found no differences in sleep duration between oncology patients and their FCs^26^. According to the standard of 420 minutes proposed by Buysse *et al*.^29^, our result indicates insufficient duration of sleep among these populations.

It should be noted that the vast majority of patients and their caregivers in this study had never used sleep medication. Only 6.0% of patient and 4.0% of FCs had ever taken medicine, a non-significant difference. These percentages, however, are slightly lower than the 17% rate of sleep medication use reported by a study of FCs of patients with advanced cancer^30^. A possible reason for the higher rate in that study is that FCs report more caregiving burden and sleep disturbance (72%) when caring for patients with a variety of cancer diagnoses or advanced cancer. Although a previous study of oncology patients and their FCs showed that patients had higher scores for use of sleep medication than FCs^26^, most FCs did not take medicine to treat their sleep problems, which is similar to previous reports^24,31^. The reluctance to use sleeping medications may be because FCs were worried about the adverse effects of the medicine or because these medications could interfere with their ability to perform caregiving duties at night. Additional reasons can be found in the results from a previous study in which 35% of patients thought that the sleep problem was not particularly important, 30% thought that sleep disturbance may be transient, and 11% did not experience consequences of the disturbance such as daily weakness^32^.

Regarding the risk factors influencing poor sleep quality in patients with NPC prior to the initiation of treatment, the current study found that age was a predictor of sleep disturbance before treatment. Older patients were more likely to suffer from worse sleep. This finding is consistent with a previous study that reported a greater prevalence of poor sleep in older adults than in younger adults^33^. However, in contrast to prior findings with cancer patients, several studies have reported that younger age was associated with sleep disturbance^19,34,35^. Moreover, one study of patients with NPC even found that age did not correlate with poor sleep quality^18^. These inconsistent results may be due to the cancer type under investigation or the time point of the measurement. Our study evaluated the quality of sleep in patients with NPC prior to the initiation of treatment. Another reason may be that elderly patients have lower expectations related to this symptom and consider sleep disturbance part of the normal ageing process.

This study also found that patients with a lower BMI were more prone to suffer from worse sleep before treatment. However, contrary to our result, Dhruva *et al*.^20^ reported that a higher BMI in patients with breast cancer was correlated with more objective sleep disturbance at the initiation of RT. This contradictory finding may be because 30% of the patients in their study had a BMI of more than 30, which is the cut-off score for obesity, whereas no patients’ BMI score exceeded this level in our study. Obesity is associated with sleep disorders^36^. Of note, inconsistent results from studies of risk factors for sleep disturbance have not found a correlation between BMI and sleep disturbance^21,37^.

Although the current study did not identify any association between education and sleep disturbance in patients with NPC before treatment, a previous study reported that education was positively associated with an improvement in sleep quality among patients with poor sleep before breast cancer treatments^38^. Furthermore, less education was a significant predictor for poorer overall sleep in oncology patients at the initiation of chemotherapy^39^. The exact factors responsible for poor sleep in patients with less education are not clear. These inconsistent findings of the relation among age, BMI and education and sleep quality indicate that further investigation is needed in future.

Several limitations of this study need to be noted. First, the current study only evaluates the quality of sleep of patients before treatment. In addition, the cross-sectional study design does not allow an evaluation of how the sleep changes over time. Second, certain demographic characteristics of the patients and FCs do not match. The majority of the FCs were younger and female and had high levels of KPS. Therefore, differences in the results of patients and FCs should be interpreted with caution. Finally, in this study, factors that predicted poor sleep quality of patients with NPC prior to the initiation of treatment only included demographic and personal characteristics and cancer-specific variables. However, a great number of previous studies have shown that pain, depression, anxiety, fatigue, and cognitive function were all associated with sleep quality in cancer patients^38,40,41^. Furthermore, these symptoms seldom occur in isolation. Rather, they often appear as symptom clusters^42,43^, a term describing three or more concurrent symptoms that are related to each other. We did not investigate these symptoms as a group in the current study, although they should be considered in future studies.

Despite these limitations, findings from this study suggest that sleep disturbance is a significant problem in patients with NPC and their FCs prior to the initiation of treatment. Moreover, patients who are older and have a lower BMI appear to be more inclined to suffer from poor sleep quality before treatment. Longitudinal studies are warranted to determine the cause-and-effect relationships between sleep disturbance before treatment and demographics, personal characteristics, and cancer-specific variables and among symptoms of pain, depression, anxiety, fatigue, cognitive function and sleep disturbance. In addition, patterns of change in sleep disturbance in patients with NPC during and after treatment should be investigated. These types of studies can provide information to design and test targeted interventions for patients with NPC who experience sleep disturbance.

## Methods

### Participants and Settings

Patients who were diagnosed pathologically with NPC were recruited to participate in this cross-sectional study in the Department of Radiation Oncology at the Cancer Hospital of Guangxi Medical University from July 2016 to April 2017. Patients were eligible for study participation if they met the following criteria: (1) diagnosis with NPC (stage I to IVb, seventh edition Union for International Cancer Control); (2) aged from 18 to 70 years; (3) gave written informed consent; and (4) had a KPS of ≥60. Patients were excluded if they had metastatic disease, more than one cancer diagnosis, had a diagnosed with sleep disorder (e.g., sleep apnea, narcolepsy, restless leg syndrome), the administration of RT or chemotherapy before cancer treatment, presence of psychiatric disorder(eg, psychosis), intellectual disability or dementia, presence of severe visual, hearing, or language defects. FCs were eligible to participate if they meet the following criteria: (1) an adult (from 18 to 70 years); (2) KPS score of greater than 60; (3) identified by the patient as the one who mostly involved in his/her actual care. FCs were excluded if they were diagnosed with sleep disorder, presence of psychiatric disorder (eg, psychosis), intellectual disability or dementia, presence of severe visual, hearing, or language defects. Prior to participation, written informed consent was obtained in accordance with a protocol that was approved by the Cancer Hospital of Guangxi Medical University, Institutional Review Board.

### Instruments

The PSQI is designed to evaluate the subjective quality of sleep in the past month. It contains 19 self-rated questions, including seven subscale components (ie, subjective sleep quality, sleep latency, sleep duration, habitual sleep efficiency, sleep disturbance, use of sleep medication, and daytime dysfunction). The global PSQI score ranges from 0 (no difficulty) to 21 (severe difficulties in all areas). Each component score ranges from 0 (no difficulty) to 3 (severe difficulty). A global PSQI score of >5 indicates a significant level of sleep disturbance. Higher global and component scores indicate more severe complaints and a higher level of sleep disturbance. However, previous validation studies have suggested a cut-off of the Chinese version global score at >7 for the presence of sleep disturbance in cancer patients^44,45^. The PSQI had a Cronbach’s α of 0.79 and displayed good test-retest reliability (r = 0.79)^46^. Therefore, in this study, a PSQI score >7 is defined as sleep disturbance, poor sleep quality or sleep disorder^47^.

### Study Procedures

Patients and FCs were approached by a trained nurse to discuss study participation and to administer the questionnaire. The details of the study were explained to each patient and their FCs. After patients gave their written informed consent, they were asked to complete the baseline study questionnaires. The total assessment lasted approximately ten minutes. Baseline questionnaires were completed prior to the initiation of treatment.

### Statistical analyses

Descriptive statistics and frequency distributions were generated for sample characteristics. The differences of demographic and clinical characteristics between patients and their FCs were compared by using Chi Square analyses or paired t-tests. Dyadic differences of PSQI were evaluated by using Chi Square analyses. Spearman correlation analysis was used to analyse correlations between global PSQI score and subscores and their demographic and clinical characteristics. Point-biserial correlations were used to analyze the nominal data. As mentioned in the instrument before, a PSQI score >7 was defined as sleep disturbance, poor sleep quality or sleep disorder. Logistic regression models were used to analyse the relationships between variables (age, BMI, gender, KPS, education level, marital status, and clinical stage) and poor sleep quality prior to the initiation of treatment. All tests were two-sided, and a p-value of < 0.05 was considered statistically significant. SPSS 16.0 (SPSS Inc., Chicago, IL, USA) was used for all analyses.

## References

[1] Torre LA (2015). Global cancer statistics, 2012. CA Cancer J Clin..

[2] Yang L (2015). Development and External Validation of Nomograms for Predicting Survival in Nasopharyngeal Carcinoma Patients after Definitive Radiotherapy. Sci Rep..

[3] Van Onselen C (2010). Relationship between mood disturbance and sleep quality in oncology outpatients at the initiation of radiation therapy. Eur J Oncol Nurs..

[4] Miaskowski C (2011). Sleep-wake circadian activity rhythm parameters and fatigue in oncology patients before the initiation of radiation therapy. Cancer Nurs..

[5] Cao XL (2017). The prevalence of insomnia in the general population in China: A meta-analysis. PLoS One..

[6] Xiao C (2016). A prospective study of quality of life in breast cancer patients undergoing radiation therapy. Adv Radiat Oncol..

[7] Lindviksmoen G, Hofso K, Paul SM, Miaskowski C, Rustoen T (2013). Predictors of initial levels and trajectories of depressive symptoms in women with breast cancer undergoing radiation therapy. Cancer Nurs..

[8] Cheng KK, Lee DT (2011). Effects of pain, fatigue, insomnia, and mood disturbance on functional status and quality of life of elderly patients with cancer. Crit Rev Oncol Hematol..

[9] Innominato PF (2015). Subjective sleep and overall survival in chemotherapy-naive patients with metastatic colorectal cancer. Sleep Med..

[10] Fletcher BS (2008). Prevalence, severity, and impact of symptoms on female family caregivers of patients at the initiation of radiation therapy for prostate cancer. J Clin Oncol..

[11] Lee KC, Yiin JJ, Lin PC, Lu SH (2015). Sleep disturbances and related factors among family caregivers of patients with advanced cancer. Psychooncology..

[12] Carter PA, Acton GJ (2006). Personality and coping: predictors of depression and sleep problems among caregivers of individuals who have cancer. J Gerontol Nurs..

[13] Chang EW, Tsai YY, Chang TW, Tsao CJ (2007). Quality of sleep and quality of life in caregivers of breast cancer patient. Psychooncology..

[14] Akman T, Yavuzsen T, Sevgen Z, Ellidokuz H, Yilmaz AU (2015). Evaluation of sleep disorders in cancer patients based on Pittsburgh Sleep Quality Index. Eur J Cancer Care (Engl)..

[15] Moses FM, Buscemi JH (1981). Obstructive sleep apnea syndrome associated with nasopharyngeal carcinoma. West J Med..

[16] Lin HC (2014). Impact of head and neck radiotherapy for patients with nasopharyngeal carcinoma on sleep-related breathing disorders. JAMA Otolaryngol Head Neck Surg..

[17] Qin L (2015). Sleep characteristics and psychological symptoms in patients with locally advanced nasopharyngeal carcinoma before and after intensity-modulated radiotherapy and concurrent chemotherapy. Psychol Health Med..

[18] Mo YL (2014). Cognitive function, mood, and sleep quality in patients treated with intensity-modulated radiation therapy for nasopharyngeal cancer: a prospective study. Psychooncology..

[19] Rogers LQ (2008). Factors associated with fatigue, sleep, and cognitive function among patients with head and neck cancer. Head Neck..

[20] Dhruva A (2012). A longitudinal study of measures of objective and subjective sleep disturbance in patients with breast cancer before, during, and after radiation therapy. J Pain Symptom Manage..

[21] Bardwell WA (2008). The relative importance of specific risk factors for insomnia in women treated for early-stage breast cancer. Psychooncology..

[22] Ancoli-Israel S, Moore PJ, Jones V (2001). The relationship between fatigue and sleep in cancer patients: a review. Eur J Cancer Care (Engl)..

[23] Lee K, Cho M, Miaskowski C, Dodd M (2004). Impaired sleep and rhythms in persons with cancer. Sleep Med Rev..

[24] Aslan O, Sanisoglu Y, Akyol M, Yetkin S (2009). Quality of sleep in Turkish family caregivers of cancer patients. Cancer Nurs..

[25] Kotronoulas G, Wengstrom Y, Kearney N (2016). Alterations and Interdependence in Self-Reported Sleep-Wake Parameters of Patient-Caregiver Dyads During Adjuvant Chemotherapy for Breast Cancer. Oncol Nurs Forum..

[26] Carney S (2011). Differences in sleep disturbance parameters between oncology outpatients and their family caregivers. J Clin Oncol..

[27] Carter PA, Chang BL (2000). Sleep and depression in cancer caregivers. Cancer Nurs..

[28] Morris BA (2015). Sleep disturbance in cancer patients and caregivers who contact telephone-based help services. Support Care Cancer..

[29] Buysse DJ, Reynolds CF, Monk TH, Berman SR, Kupfer DJ (1989). The Pittsburgh Sleep Quality Index: a new instrument for psychiatric practice and research. Psychiatry Res..

[30] Lee KC, Yiin JJ, Lu SH, Chao YF (2015). The Burden of Caregiving and Sleep Disturbance Among Family Caregivers of Advanced Cancer Patients. Cancer Nurs..

[31] Gibbins J (2009). Sleep-wake disturbances in patients with advanced cancer and their family carers. J Pain Symptom Manage..

[32] Romito F (2014). Patients attitudes towards sleep disturbances during chemotherapy. Eur J Cancer Care (Engl)..

[33] Ancoli-Israel S (2009). Sleep and its disorders in aging populations. Sleep Med..

[34] Miaskowski C (2011). Predictors of the trajectories of self-reported sleep disturbance in men with prostate cancer during and following radiation therapy. Sleep..

[35] Palesh OG (2010). Prevalence, demographics, and psychological associations of sleep disruption in patients with cancer: University of Rochester Cancer Center-Community Clinical Oncology Program. J Clin Oncol..

[36] Desalu OO (2017). Identifying patients at high risk for obstructive sleep apnoea syndrome in Nigeria: A multicentre observational study. Malawi Med J..

[37] Sivertsen B, Krokstad S, Overland S, Mykletun A (2009). The epidemiology of insomnia: associations with physical and mental health. The HUNT-2 study. J Psychosom Res..

[38] Fontes Filipa, Pereira Susana, Costa Ana Rute, Gonçalves Marta, Lunet Nuno (2017). The impact of breast cancer treatments on sleep quality 1 year after cancer diagnosis. Supportive Care in Cancer.

[39] Phillips KM, Jim HS, Donovan KA, Pinder-Schenck MC, Jacobsen PB (2012). Characteristics and correlates of sleep disturbances in cancer patients. Support Care Cancer..

[40] Stepanski EJ (2009). The relation of trouble sleeping, depressed mood, pain, and fatigue in patients with cancer. J Clin Sleep Med..

[41] Chen ML, Yu CT, Yang CH (2008). Sleep disturbances and quality of life in lung cancer patients undergoing chemotherapy. Lung Cancer..

[42] Ho SY, Rohan KJ, Parent J, Tager FA, McKinley PS (2015). A longitudinal study of depression, fatigue, and sleep disturbances as a symptom cluster in women with breast cancer. J Pain Symptom Manage..

[43] Lin S, Chen Y, Yang L, Zhou J (2013). Pain, fatigue, disturbed sleep and distress comprised a symptom cluster that related to quality of life and functional status of lung cancer surgery patients. J Clin Nurs..

[44] Tzeng JI, Fu YW, Lin CC (2012). Validity and reliability of the Taiwanese version of the Pittsburgh Sleep Quality Index in cancer patients. Int J Nurs Stud..

[45] Carpenter JS, Andrykowski MA (1998). Psychometric evaluation of the Pittsburgh Sleep Quality Index. J Psychosom Res..

[46] Ho RT, Fong TC (2014). Factor structure of the Chinese version of the Pittsburgh sleep quality index in breast cancer patients. Sleep Med..

[47] Sun D, Shao H, Li C, Tao M (2014). Sleep disturbance and correlates in menopausal women in Shanghai. J Psychosom Res..

